# Cholera Asiatica

**Published:** 1911-08

**Authors:** 


### Cholera Asiatica

THE American epidemics of cholera occurred in 1832, lasting till 1834, 1848, 1854—mute remains of which were turned out of the old Masten Park Cemetery a few years ago—, 1865-'6, and 1873, limited in the last year to New Orleans and the Mississippi Valley and neighboring states. The history of cholera in Europe goes back to the fathers of medicine and includes many devastating epidemics. For centuries, cholera was believed to follow the foot steps of the Wandering Jew, the legend undoubtedly being an attempt to explain the curious deviations in the route of cholera and the skipping of cities and localities which would have been involved by an air-borne epidemic. Religion has more to do with the spread of cholera than the invention of a legend. The cholera spirillum seems to have evolved as a specific pathogen in Asia, probably in India. The belief in the sacredness of the Ganges has led to a continued, almost continuous infection of its waters, by bathing, as well as by natural drainage from fomites. Visitors from a distance, drinking and bathing in its waters and carrying the precious liquid back to their home have maintained an endemicity of the disease independently of and even against natural currents. On the other hand, pilgrimages of the Mohametan portion of the Indian population have transferred the infection to the equally holy well at Mecca whence, both by religious and commercial intercourse, the disease has been still further radiated.

The Wandering Jew has often knocked at our door. In 1892, another American epidemic was greatly feared but vigorous quarantine measures prevented a lodgement. This summer, cholera may be said to have effected a foothold. At present writing, as late as our date of publication admits, nearly twenty cases have been treated, about half having died. Two resident cases have developed, one in New York, the other in Boston. While we are not apprehensive of anything approaching an epidemic, some degree of alarm is excusable and salutary. Cholera and typhoid are the two prominent hydrophoric—water-borne—infections, if indeed they are not the only ones of this essential etiology with which European and American physicians have to deal. In temperate and cold climates, cholera epidemics have usually subsided quite promptly, without becoming endemic even in the sense of typhoid. Apparently, therefore, the cholera spirillum is a delicate organism, not capable of withstanding ordinary natural, extra-corporeal vicissitudes. But it is a disease of approximately 50 per cent. mortality, as compared with about 10 per cent. for typhoid and it is said not to be semelincident, i. e., one attack does not confer immunity. The authorities have been severely blamed for not having jugulated the disease at its first appearance. Censure should be withheld until a thorough investigation shall have been made. Meantime, vigorous quarantine and disinfecting measures are being carried out and we hope that no further development will occur.

